# Case of Gun-Shot Injury of the Tibia

**Published:** 1872-10

**Authors:** Charles P. Gordon

**Affiliations:** Dalton, Georgia


### CASE OF GUN-SHOT INJURY OF THE TIBIA.

BY CHARLES P. GORDON, M.D., DALTON, GEORGIA.

Colonel J. A. W. Johnson, of this place, received a gun-shot injury of the tibia at the battle of Missionary Ridge, November 25th, 1863, the ball impinging upon the left tibia, four or five inches below the head of the bone, at its antero-lateral aspect, penetrating into and lodging within the medullary canal. The patient being of the opinion that he had himself extracted the ball with some piecees of clothing driven into the wound at the time of injury, the surgeons in attendance made no thorough examination for the ball, and the patient was forwarded to the hospital at Macon, Georgia. The wound not healing in due time, an operation was deemed necessary for the removal of necrosed bone, and was accordingly performed by Dr. G. F. Cooper, surgeon in charge of the hospital, some time in July or August, 1864, seven or eight months after the receipt of injury. When he came under my charge, subsequent to the war, the circumference of the bone was found greatly enlarged by hypertrophy, at the point of injury—the entrance of the ball being obliterated by the formation of new bone, with a single cloaca leading down to the medullary canal, discharging pus, and sometimes attended with copious hemorrhage. Upon an examination of the wound with the probe, was discovered what was supposed to be a sequestrum—the patient still affirming that he had himself extracted the ball at the time of injury, and an operation was instituted for its removal, June 1st, 1867. The patient being thoroughly under the influence of chloroform, a crucial incision was made, and flaps dissected up to the periosteum, which being sufficiently removed, a large trephine was applied, the shaft of which was soon twisted in twain, such was the extreme density and ivory hardness of the bone; fortunately, another being on hand, it was applied, and a circle of bone of great density and thickness was removed down to the medullary canal, when a large minnie ball was discovered firmly impacted in the canal, which being removed, and the cavity scraped out with the gouge, the wound being brought together with sutures and adhesive plaster, the patient made a speedy and permanent recovery.

Attention is called to the fact, that in this case the ball remained in the medullary canal of the tibia four years and seven months, without producing caries or necrosis of the bone—the only pathological results attending its presence being a hypertrophic enlargement of the circumference of the bone, with the discharge of pus and blood from the wound. The constitution of the patient bore the severe operation without the slighest resentment, and, as above stated, he made a most favorable recovery.

